# The role of the gut-microbiome-brain axis in metabolic remodeling amongst children with cerebral palsy and epilepsy

**DOI:** 10.3389/fneur.2023.1109469

**Published:** 2023-02-27

**Authors:** Ye Peng, Annie T. G. Chiu, Vivien W. Y. Li, Xi Zhang, Wai L. Yeung, Sophelia H. S. Chan, Hein M. Tun

**Affiliations:** ^1^The Jockey Club School of Public Health and Primary Care, Faculty of Medicine, The Chinese University of Hong Kong, Shatin, Hong Kong SAR, China; ^2^Microbiota I-Center (MagIC), The Chinese University of Hong Kong, Shatin, Hong Kong SAR, China; ^3^Li Ka Shing Institute of Health Sciences, Faculty of Medicine, The Chinese University of Hong Kong, Shatin, Hong Kong SAR, China; ^4^Department of Paediatrics and Adolescent Medicine, Hong Kong Children's Hospital, Kowloon City, Hong Kong SAR, China; ^5^Department of Paediatrics and Adolescent Medicine, Queen Mary Hospital and Duchess of Kent Children's Hospital, Pokfulam, Hong Kong SAR, China; ^6^Department of Paediatrics and Adolescent Medicine, School of Clinical Medicine, Li Ka Shing Faculty of Medicine, The University of Hong Kong, Pokfulam, Hong Kong SAR, China

**Keywords:** epilepsy, cerebral palsy, gut-brain axis, gut microbiome, gut metabolome, drug resistant epilepsy, multi-omics

## Abstract

**Background:**

Epilepsy-associated dysbiosis in gut microbiota has been previously described, but the mechanistic roles of the gut microbiome in epileptogenesis among children with cerebral palsy (CP) have yet to be illustrated.

**Methods:**

Using shotgun metagenomic sequencing coupled with untargeted metabolomics analysis, this observational study compared the gut microbiome and metabolome of eight children with non-epileptic cerebral palsy (NECP) to those of 13 children with cerebral palsy with epilepsy (CPE). Among children with CPE, 8 had drug-sensitive epilepsy (DSE) and five had drug-resistant epilepsy (DRE). Characteristics at enrollment, medication history, and 7-day dietary intake were compared between groups.

**Results:**

At the species level, CPE subjects had significantly lower abundances of *Bacteroides fragilis* and *Dialister invisus* but higher abundances of *Phascolarctobacterium faecium* and *Eubacterium limosum*. By contrast, DRE subjects had a significantly higher colonization of *Veillonella parvula*. Regarding microbial functional pathways, CPE subjects had decreased abundances of pathways for serine degradation, quinolinic acid degradation, glutamate degradation I, glycerol degradation, sulfate reduction, and nitrate reduction but increased abundances of pathways related to ethanol production. As for metabolites, CPE subjects had higher concentrations of kynurenic acid, 2-oxindole, dopamine, 2-hydroxyphenyalanine, 3,4–dihydroxyphenylglycol, L-tartaric acid, and D-saccharic acid; DRE subjects had increased concentrations of indole and homovanilic acid.

**Conclusions:**

In this study, we found evidence of gut dysbiosis amongst children with cerebral palsy and epilepsy in terms of gut microbiota species, functional pathways, and metabolites. The combined metagenomic and metabolomic analyses have shed insights on the potential roles of *B. fragilis* and *D. invisus* in neuroprotection. The combined analyses have also provided evidence for the involvement of GMBA in the epilepsy-related dysbiosis of kynurenine, serotonin, and dopamine pathways and their complex interplay with neuroimmune and neuroendocrinological pathways.

## Introduction

Cerebral palsy (CP) refers to “a group of permanent, but not unchanging, disorders of movement and/or posture and of motor function, which are due to non-progressive interference, lesion, or abnormality of the developing brain” (1). The specific area of the brain affected in cerebral palsy is variable and depends on the timing, mechanism, and severity of the insult. Premature babies of 32 weeks or younger are prone to periventricular leukomalacia, whereas full term babies with hypoxic ischemic encephalopathy are prone to having lesions over the deep gray structures. In more severe cases the cortical, subcortical, and brainstem structures are also affected. CP is one of the most common causes of neurological disability in children and up to 15–55% of children with CP have comorbid epilepsy (2), the risk of which increases with higher grades of motor function deficit (3, 4).

The gut microbiota-brain axis (GMBA) is a bi-directional communication network where signals derived from gut microbiota can affect the central nervous system, enteric nervous system, autonomic nervous system, neuro-endocrine system, and neuro-immune system. Dysbiosis of gut microbiota has been implicated in acute phases of traumatic brain injury (5) amongst children with cerebral palsy (6). A rat model of cerebral palsy treated with *Saccharomyces boulardii* showed improvement in depression-like behavior and increased microbiota diversity (7). Children with CP and epilepsy (CPE), compared to healthy children, also exhibited significantly higher microbial diversity and different bacterial profiles in their gut microbiota (6). However, it is difficult to attribute the observed gut dysbiosis to either epilepsy, CP, or the distinct lifestyle and dietary factors related to both interactive neurological diseases.

The GMBA is also implicated in epileptogenesis. In prior mouse models, transplantation of the gut microbiome from stress donors to sham-stressed subjects led to increased seizures; the opposite was shown to ameliorate the pro-convulsive effect of chronic stress (8). Another mouse model also demonstrated that the transplantation of gut microbiota from either mice responding to ketogenic diet (KD) or bacterial species associated with KD (*Akkamensia* and *Parabacteroides)* conferred seizure protection to mice fed a control diet (9, 10). A study comparing the gut microbiota of patients with drug resistant epilepsy (DRE) to those with drug sensitive epilepsy (DSE) and healthy controls showed that, in DRE patients, the gut microbiome is significantly altered, with increased abundances of rare flora (11). This suggests that microbial dysbiosis may be involved in the mechanism of DRE and that the restoration of the gut microbiome may be a potential therapeutic target for DRE. Previous clinical studies also demonstrated that there are differential circulating metabolites in patients with epilepsy, especially those with DRE (12–14).

GMBA involves a complex interplay of neurotransmitters and neuroimmune and neuroendocrinological modulation. However, the mechanism by which gut dysbiosis leads to neuromodulation and epileptogenesis remains to be well-understood. In this study, we aim to elucidate the mechanistic roles of the gut microbiome in epileptogenesis following cerebral palsy and identify gut microbiota alterations related to seizure control. This cross-sectional study used shotgun metagenomic sequencing coupled with untargeted metabolomics analysis of the gut microbiome of children with CP with or without epilepsy.

## Materials and methods

### Study design

This study was approved by the Hong Kong West Cluster Institutional Review Board (UW 21-029). We recruited participants from the Pediatric Neurology Out-Patient Clinics and the Pediatric Wards of the Department of Pediatrics and Adolescent Medicine, Queen Mary Hospital and Duchess of Kent Children's Hospital, Hong Kong. Children aged 1–16 years old, who were diagnosed with CP, were recruited with written informed consent obtained from parents or legal guardians. Children with CPE were diagnosed according to definitions laid down by the International League Against Epilepsy (15) and categorized as either DSE or DRE according to the definitions published by Kwan et al. (16). Children who had a known history of gastrointestinal disorders, who were on gastrostomy feeding, who had coexisting neurometabolic or metabolic conditions, had recent uses of oral antibiotics within 1 month, or had recent travel history outside of Hong Kong, were excluded.

### Data and sample collection

Medical information of all the recruited patients were systematically collected from health records. Carers were asked to record a 7-day dietary intake by the subjects using a Food Frequency Questionnaire which was previously adopted and validated by the Government of the Hong Kong Special Administrative Region in the city's first population-based food survey (17). Carers were also asked to document the consistency of subjects' stool using the Bristol stool scale. Fecal samples were collected using the OMNIgene•GUT kit (DNA Genotek) at home or the hospital and transferred to the laboratory within a week for storage. Upon reception at the laboratory, samples were kept at −80°C until further analyses.

### Metagenomic analysis

DNA was extracted using the QIAamp PowerFecal Pro DNA Kit (QIAGEN), followed by shotgun metagenomic sequencing (150-bp paired-end) at DNBseq^TM^ sequencing platform. Reads with low quality or mapped to the human reference genome (hg38) were removed. Taxa and functional pathways were profiled by Humann3 and Omixer-rpm, respectively. Observed species, Shannon diversity index, and Bray-Curtis dissimilarity were calculated based on the bacterial species profile.

### Metabolomic analysis

Metabolites were extracted from ~80 mg of each fecal sample. Aliquots from each prepared sample were mixed into quality control (QC) samples. Metabolites were separated using a Waters ACQUITY UPLC BEH C18 column (Waters) in both positive-ion and negative-ion modes and analyzed by a Q-Exactive mass spectrometer (Thermo Fisher Scientific). Data processing and metabolite identification were done by Compound Discoverer 3.1 based on the KEGG, mzCloud, and HMDB databases. The data were then normalized using the Probabilistic Quotient Normalization, corrected for batch effect by QC sample-based robust LOESS signal correction, and filtered by removing compounds with a Coefficient of Variation >30% of the relative peak area in all QC samples.

### Statistical methods

Continuous and categorical data between groups were compared using Wilcoxon rank-sum test and Fisher's exact test, respectively. Beta diversity was compared using *adonis* test. Differential metabolites were identified by *t*-test (*p* < 0.05), fold change (FC) analysis (FC < 0.833 or >1.2), and partial least squares discriminant analysis (a variable importance >1). Generalized linear models were constructed to model associations between species abundance while adjusting for confounders where appropriate. All statistical analyses were performed in R (v4.1.0).

## Results

### A cohort of pediatric cerebral palsy patients

A total of 27 children with CP were recruited initially. This included 17 with epilepsy (CPE), of which 5 had DRE and 12 had DSE at enrollment. One patient born abroad with an incomplete medical record and five children whose epilepsy status changed after enrollment were subsequently excluded. This resulted in eight children with NECP and 13 children with CPE, of which five had DRE and eight had DSE. Baseline characteristics including age, GMFCS score, length of stay in neonatal care unit, duration of antibiotic exposure within the first 3 months of life, previous use of gut motility and gastric acid suppressing medications, bowel habit, stool consistency, and probiotics use, were not statistically significant between the CPE and NECP groups, nor between DSE and DRE. Compared to the DRE subgroup, the DSE subgroup consumed fewer vegetables but more fish (Table 1).

**Table 1:** Characteristics and 7-day diet history of the cerebral palsy patients recruited in this study.

**Characteristic**	**NECP**	**CPE**	**DSE**	**DRE**	***P*** **-value**	
	**(*****N*** = **8)**	**(*****N*** = **13)**	**(*****N*** = **8)**	**(*****N*** = **5)**	**NECP vs. CPE**	**DRE vs. DSE**
Age [years, median (range)]	5.4 (1.9–9.5)	8.8 (4.5–16.9)	8.0 (4.8–16.8)	9.7 (4.5–15.4)	0.060	0.489
Sex					0.659	0.103
Female	5 (62.5)	6 (46.2)	2 (25)	4 (80)		
Male	3 (37.5)	7 (53.8)	6 (75)	1 (20)		
Type of cerebral palsy					0.377	1
Spastic hemiplegic	0 (0.0)	1 (7.7)	1 (12.5)	0 (0.0)		
Spastic diplegic	2 (25.0)	3 (23.1)	2 (25.0)	1 (20.0)		
Spastic triplegic	3 (37.5)	1 (7.7)	1 (12.5)	0 (0.0)		
Spastic quadriplegic	3 (37.5)	8 (61.5)	4 (50.0)	4 (80.0)		
GMFCS class					0.278	0.394
I	0 (0.0)	3 (23.1)	3 (37.5)	0 (0.0)		
II	2 (25.0)	1 (7.7)	0 (0.0)	1 (20.0)		
III	3 (37.5)	1 (7.7)	1 (12.5)	0 (0.0)		
IV	2 (25.0)	4 (30.8)	2 (25.0)	2 (40.0)		
V	1 (12.5)	4 (30.8)	2 (25.0)	2 (40.0)		
Number of AEDs used (at the time of enrollment)					0	0.032
0	8 (100.0)	0 (0.0)	0 (0.0)	0 (0.0)		
1	0 (0.0)	8 (61.5)	7 (87.5)	1 (20.0)		
Epilim	0	6	5	1^†^		
Keppra	0	1	1	0		
Topiramate	0	1	1	0		
2	0 (0.0)	5 (38.5)	1 (12.5)	4 (80.0)		
Epilim + Nitrazepam	0	1	1	0		
Epilim + Vigabatrin	0	1	0	1		
Epilim + Keppra	0	1	0	1		
Keppra + Vigabatrin	0	1	0	1		
Keppra + Clobazam	0	1	0	1		
Enzyme inhibitors use^‡^	0 (0)	9 (69.2)	6 (75.0)	3 (60.0)	0.005	1
Bowel habit					0.289	0.51
Less than every 2 days	1 (12.5)	3 (23.1)	1 (12.5)	2 (40.0)		
Daily or every 2 days	5 (62.5)	10 (76.9)	7 (87.5)	3 (60.0)		
More than once per day	2 (25.0)	0 (0.0)	0 (0.0)	0 (0.0)		
Bristol scale (7)	3 (2, 4)	3 (2, 4)	3 (2.5, 4)	3 (2, 4)	1	0.462
Probiotics use^§^	0 (0.0)	2 (15.4)	2 (25.0)	0 (0.0)	0.505	0.487
**7-day dietary intake (number of portions consumed per week)** ^¶^						
Cereal	12 (9.6, 14)	7 (7, 14)	10 (6, 14)	7 (7, 14)	0.432	0.433
Vegetables	7 (6.1, 14)	7 (7, 10)	7 (3.4, 7.9)	7 (7, 21)	0.791	**0.046**
Fruit	7 (4.2, 7)	6 (1.5, 7)	4.5 (1.2, 7)	7 (3.5, 7)	0.319	0.273
Meat/poultry	6 (4.6, 7)	7 (6, 7)	7 (6, 14)	7 (7, 7)	0.288	0.263
Eggs products	6 (3.9, 8.8)	3 (1, 6)	4 (2.8, 6.2)	0.5 (0, 2)	0.243	0.067
Fish	3.5 (1.9, 4)	4 (2, 7)	5 (3.5, 7)	2 (1, 3)	0.380	**0.027**
Other seafood	0.31 (0, 2.2)	0 (0, 0.5)	0.25 (0, 0.62)	0 (0, 0)	0.240	0.198
Beans/nuts products	1 (0.75, 1.2)	2 (0, 4)	1.5 (0, 3.2)	3.5 (1, 7)	0.458	0.163
Dairy products	7 (5.8, 8.8)	7 (2, 14)	6.5 (2, 12)	7 (7, 14)	1	0.257
Sugary drinks	0 (0, 0.062)	0 (0, 0)	0 (0, 1.9)	0 (0, 0)	0.961	0.057

GMFCS, Gross motor function classification system; AED. Antiepileptics; DRE, Drug resistant epilepsy; DSE, Drug sensitive epilepsy; GI, Gastrointestinal medications including laxatives, antacids, etc.

† This DRE patient had previously tried other AEDs including Nitrazepam/Clonaxepam/Vigabatrin, but later took only Epilim with better seizure control. This fulfills the definition of DRE, i.e., sequential or concomitant trial of appropriately chosen ≥2 AED in good dose.

‡ Sodium valproate was the only enzyme inhibitor used in this study. None of the subjects used enzyme inducers (e.g., phenytoin, phenobarbitone, carbamazepine).

§ One patient used 2 types of probiotics: BIoGaia, ProTectis baby drops (*Lactobacillus reuteri*) & Dr Ohhira's, Probiotics (*Bifodobacteria, Enterococcus faecalis, Streptoccocus thermophilus*); the other patient used Catalo Children's probiotics formula, including *Lactobacillus acidophilus, Bifidobacterium lactics*, and Fructtooligosaccharides (FOS).

¶ Total size of food, calculated by frequency × size per meal. Categorical data were presented as number (percentage). Continuous data were shown as median (25th quantile, 75th quantile) unless specified. P values for comparisons of categorical data and continuous data were given by Fisher's exact tests and Wilcoxon rank-sum tests, respectively.

### Gut microbiome in children with cerebral palsy and epilepsy

**Figure 1:**
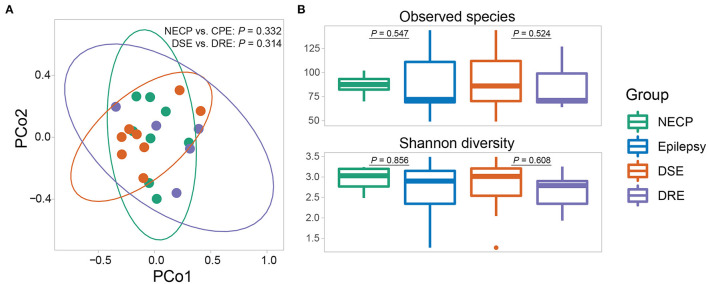
**(A)** Beta diversity (based on Bray-Curtis dissimilarity) of the gut microbial communities. *P*-values were given by adonis tests. **(B)** Alpha diversity (observed species and Shannon diversity) of the gut microbial communities. *P*-values were given by Wilcoxon rank-sum tests.

**Figure 2:**
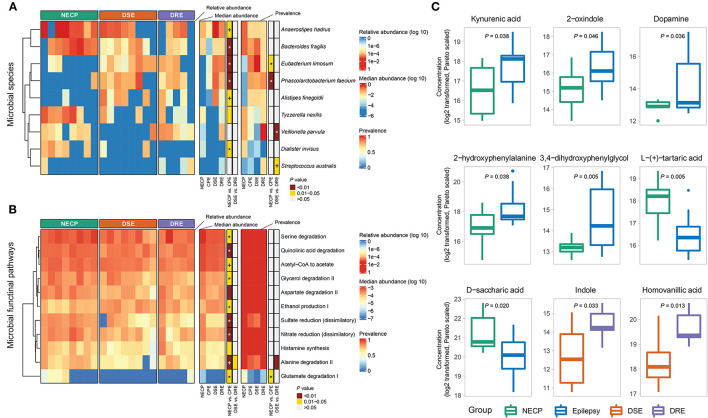
**(A)** Distributions of differentially abundant/prevalent species between groups. **(B)** Distributions of differentially abundant/prevalent functional pathways between groups. **(C)** Selected differentially abundant metabolites between NECP and CPE and between DSE and DRE. *P*-values were categorized into “ <0.01,” “0.01–0.05,” and “>0.05” and indicated by colors. Those that were significantly associated with the group (NECP vs. CPE or DSE vs. DRE) in the generalized linear model were denoted by “*” (*P* < 0.05) and “+” (0.05 < *P* < 0.1). Models for NECP vs. CPE were adjusted for age, whereas models for DSE vs. DRE were adjusted for sex. Values were log2-transformed and pareto-normalized. *P*-values were given by independent *t*-tests.

Overall microbial compositions were not different between subgroups (Figure 1A) and were not associated with environmental factors (Supplementary Table 1). There was no significant difference in alpha diversity (observed species and Shannon diversity indices) between the CPE and NECP groups, nor between the DSE and DRE subgroups for children with CPE (Figure 1B). At the species level, compared to the NECP group, children with CPE had significantly lower abundances of *Bacteroides fragilis* and *Dialister invisus*, but higher abundances of *Phascolarctobacterium faecium* and *Eubacterium limosum* (Figure 2A). Within the CPE group, there was no significant difference in a specific species' abundance, but DRE subjects had a significantly higher colonization rate of *V. parvula* than DSE subjects (Figure 2A). Regarding microbiome functional pathways, CPE subjects had decreased abundances of pathways for serine degradation, quinolinic acid degradation, glycerol degradation, sulfate reduction (dissimilatory), nitrate reduction (dissimilatory), and glutamate degradation I, but increased abundances of pathways related to ethanol production I (Figure 2B). As for comparisons between DRE and DSE, the dopamine degradation and 3,4-dihydroxyphenylacetic acid (DOPAC) synthesis pathways tended to be enriched in DRE (Supplementary Table 2).

### Gut metabolome in children with cerebral palsy and epilepsy

Our metabolomic analysis showed CPE subjects had higher concentrations of kynurenic acid (NECP vs. CPE: FC = 0.40, *P* = 0.038), 2-oxindole (NECP vs. CPE: FC = 0.35, *P* = 0.046), dopamine (NECP vs. CPE: FC = 0.04, *P* = 0.036), 2-hydroxyphenyalanine (ortho-tyrosine) (NECP vs. CPE: FC = 0.28, *P* = 0.038), and 3,4–dihydroxyphenylglycol (NECP vs. CPE: FC = 0.14, *P* = 0.005). CPE subjects also had lower levels of L-tartaric acid (NECP vs. CPE: FC = 3.33, *P* = 0.005) and D-saccharic acid (NECP vs. CPE: FC = 2.50, *P* = 0.020) than their NECP counterparts. Within the CPE group, children with DRE, when compared to children with DSE, had higher concentrations of indole (DSE vs. DRE: FC = 0.31, *P* = 0.033) and homovanilic acid (DSE vs. DRE: FC = 0.40, *P* = 0.013; Figure 2C).

### Inter-omics correlations

**Figure 3:**
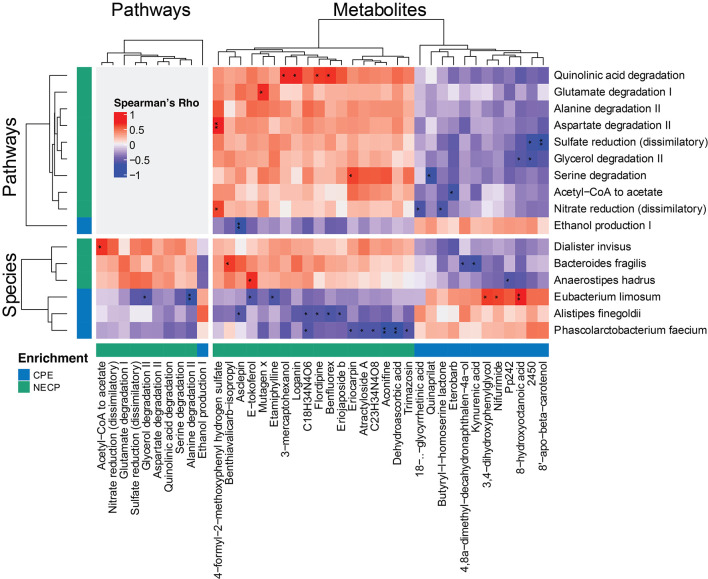
Correlations among significantly differential species, functional pathways, and metabolites between NECP and CPE. Only those having at least one correlation with an FDR-corrected *p* < 0.1 were shown. “*,” 0.05 < FDR < 0.1; “**,” 0.01 < FDR < 0.05. NECP, non-epilepsy cerebral palsy patients; CPE, cerebral palsy patients with epilepsy.

We next investigated the correlations among the differential features between CPE and NECP. *B. fragilis* was negatively correlated with kynurenic acid concentration (Rho = −0.69, *P* < 0.001, FDR = 0.058) (Figure 3). Additionally, while *E. limosum* was co-abundant with 3,4–dihydroxyphenylglycol (DHPG) (Rho = 0.67, *P* = 0.001, FDR = 0.071) and 8-hydroxyoctanoic acid (Rho = 0.74, *P* < 0.001, FDR = 0.042) (Figure 3), it was negatively correlated with ethanol production I and glycerol degradation II pathways. Furthermore, we also found that *P. faecium* was inversely correlated with dehydroascorbic acid concentration (Rho = −0.77, *P* < 0.001, FDR = 0.034). *D. invisus* was positively correlated with a pathway converting acetyl CoA to acetate (Figure 3).

## Discussion

Our study collected a special cohort of subjects that offered a unique opportunity to study the effect of the GMBA amongst a homogenous group of patients with epilepsy secondary to their cerebral palsy using a multi-omics approach. Our study reproduced findings of differentially abundant commensals and opportunistic pathogens between disease groups. Our study also yielded new insights on the neuroprotective roles of specific bacterial species (e.g., *B. fragilis* and *D. invisus*), provided evidence of epilepsy-related neurotransmitter dysbiosis and highlighted their complex interplay with neuroimmune and neuroendocrinological pathways.

### Evidence of gut dysbiosis

At the species level, *B. fragilis* was significantly increased in NECP. The correlation between *B. fragilis* abundance and non-epileptic patients is corroborated by a past study which showed that *B. fragilis* is increased amongst healthy controls compared to DRE patients. As such, there may be a protective effect of *B. fragilis* against an underlying epileptic tendency (11). Indeed, a recent pilot intervention study demonstrated a beneficial effect of *B. fragilis* in a drug resistant epilepsy group, where up to 60% of the studied patients demonstrated a more than 50% reduction in seizure frequency (18).

*D. invisus* was more abundant amongst patients with NECP. This may reflect the potential antiepileptic properties of *D. invisus*, whose abundance increases amongst DRE patients after treatment with KD (19). The mechanism by which *D. invisus* is antiepileptic is uncertain. However, *D. invisus* was correlated with a pathway converting acetyl CoA to acetate; acetate is a short chain fatty acid postulated to decrease neuroexcitability and neuroinflammation (20).

Among other differentially abundant species, the lower abundance of *P. faecium* in the NECP group concurs with a reported negative association of *P. faecium* with healthy controls (11). We also found, for the first time, an association between epilepsy and *E. limosum*, a species found more abundant in patients with autism spectrum disorder children (21, 22). As we found that *E. limosum* was co-abundant with 3,4-DPHG, *E. limosum* might play a role in the metabolic conversion of norepinephrine to 3,4–DHPG, causing a proconvulsant effect (23, 24).

Furthermore, *V. parvula* was increased in epileptic patients, particularly in DRE patients, which is consistent with a prior study (6). Interestingly, *Veillonella* was not only reported to be the most abundant genera in CPE patients (6), but it was also increased in ADHD (25) and schizophrenia patients (26). Further investigations are needed to delineate its mechanistic role in epilepsy, which is associated with both conditions (27, 28).

### Neurotransmitters pathways and their epileptogenic potential within the GMBA

Our findings unravel the kynurenine pathway as one of the potential underlying mechanisms of epilepsy amongst children with CP (Supplementary Figure 1A). CPE subjects had higher levels of kynurenic acid and a lower abundance of the pathway for the degradation of quinolinic acid, a downstream metabolite of kynurenine pathway. Kynurenic acid may be produced by the gut microbiome or through the metabolic conversion from kynurenine, an antagonist of the NMDA receptor with a neuroprotective effect (29) which is dampened in subjects with hippocampal sclerosis (30). Although we were unable to detect significant differences in the levels of kynurenine and serotonin between CPE and NECP patients, the reduction in abundance of pathways for quinolinic acid degradation in CPE patients may imply a decreased activation of the kynurenine pathway. As the kynurenine pathway also metabolizes 95% of tryptophan (31), the precursor of serotonin, this may shunt tryptophan conversion toward serotonin production. Mouse models of depression have also shown that kynurenic acid may directly activate serotonin receptors (32). Serotonin levels are raised in seizures and implicated in the pathophysiology of both sudden unexpected death in epilepsy (SUDEP) (33) and kindling, the process whereby repeatedly induced seizures results in increased seizure frequency and recruitment of neuronal circuitry (34–36). It is likely that the multi-faceted interaction between kynurenic acid, kynurenine, and serotonin plays a role in epileptogenesis amongst CPE.

CPE subjects also had higher concentrations of dopamine, ortho-tyrosine (a rare isomer of the dopamine precursor tyrosine), and 3,4-DHPG, a metabolite of dopamine and norepinephrine. By contrast, homovanilic acid, another dopamine metabolite, is more abundant amongst DRE subjects (Supplementary Figure 1B). Microbial pathways responsible for dopamine degradation and DOPAC synthesis were also enriched in DRE subjects compared to DSE subjects, possibly reflecting the increased availability of dopamine. Studies predominantly focusing on Parkinson's disease have shown that the gut microbiome is an important modulator of plasma dopamine concentrations (37). Dysbiosis of the dopamine system is well reported in epilepsy. There is an intricate balance and interaction between dopamine receptors. Taken individually, stimulation of certain dopamine receptors produces proconvulsant effect, whilst in others produce antiepileptic effects. Their respective intra-signaling pathways converge downstream, and help to regulate seizure-induced cell death and epileptogenesis (38). During seizures, dopamine levels within the cerebrospinal fluid are known to increase in both humans and animal models (39), whereas during the interictal phase, dopamine levels are lower in epileptic patients compared to non-epileptic controls (40); there is a higher expression of dopamine transporters on single-photon emission computerized tomography (SPECT) scans (41). Our finding suggests that dopamine dysbiosis in epilepsy does not confine itself within the brain but extends to the GMBA, making the GMBA a potential therapeutic target.

CPE subjects have decreased abundances of serine degradation and glutamate degradation pathway I. Both glutamate and serine are agonists of the NMDA receptor and have excitatory potential (Supplementary Figure 1C); they are implicated in epileptogenesis (42, 43). *B. fragilis* is a glutamate metabolizer (44) and could potentially influence serum glutamate level, although we found no statistically significant association between CPE and fecal glutamate concentration. We postulate that increased serine and glutamate levels and the enhanced activation of the NMDA receptor may play a role in epileptogenesis amongst CPE subjects, possibly due to reduction in *B. fragilis*.

### Complex interplay with neuroendocrinological, neuroimmune factors, and hepatic metabolism

It should be noted that complex neuroendocrinological and neuroimmune cross-talks also affects the GMBA. Dopamine mediates the stress response *via* the hypopituitary-thalamic axis and prior mouse models have shown that stress-induced seizure kindling is mediated by the gut microbiome (8).

Microbiota-deficient germ-free animals are associated with reduced expression of toll-like receptors within the gastrointestinal tract and reduced kynurenine pathway metabolism (45). Metabolites from kynurenine and indole pathways, both of which metabolize tryptophan, are also known to activate aryl hydrocarbon receptors (AHRs). AHRs are expressed on intestinal immune cells and stimulate production of IL-22, an anti-inflammatory cytokine (46). Asiatic acid levels amongst CPE were 15-fold greater than that of NECP. Asiatic acid has also been reported to have immunomodulatory effects (47).

Antiepileptics usage and their impact on liver metabolism also affects the GMBA. For instance, kynurenic acid is the target of commonly used antiseizure medications such as levetiracetam (48) and sodium valproate (49). Through the action of AHRs, kynurenine, and indole are involved in the regulation of cytochrome P450s (50), which plays a pivotal role in the hepatic metabolism of different antiseizure medications.

Of note, D-saccharic acid, which is decreased in CPE compared to NECP, has been linked to risk of neurocognitive disorders. This could possibly be attributed to disturbed carboxylate metabolism in the gut microbiome (51), although the mechanism by which this occurs is unclear.

Taken together, these findings suggest that epileptogenesis in pediatric CP patients is associated with gut microbial and metabolomic dysbiosis.

### Limitations of the study and opportunities for further study

This study had a small sample size due to low prevalence of cerebral palsy in Hong Kong. Therefore, samples collected in this pilot study prevented us from adopting commonly used methods accounting for compositionality, which either requires a larger sample size (linear discriminant analysis) or would complicate the interpretation with the resulting degenerate distribution (centered log-ratio transformation). Hence, we relied on conventionally used nonparametric tests in this study. Of course, future studies with absolute quantification are needed. A multi-center international study would therefore be needed to determine the generalizability of our findings. A longitudinal follow-up study would also be beneficial to identify the relationship between gut microbiome and epilepsy control, as well as potential epileptic syndrome-specific microbiome signatures with epileptic encephalopathy.

Furthermore, some of the results from the study remain to be validated further. For example, glycerol and sulfate do not have well-established links with epileptic activities. On the other hand, ethanol is involved in both GABA signaling and NLRP3 mediated neuroinflammation (52, 53), but whether its serum level can be significantly altered by the gut microbiome remains to be investigated. Moreover, metabolites such as ethanol and nitrate are implicated as both proconvulsant and, with apparently conflicting evidence (54–57). Therefore, studies with extensive omics data from multiple body sites and systems (e.g., cerebrospinal fluid, serum, and gut) are needed to confirm causality.

## Conclusion

Here we found specific gut bacterial species and metabolic markers associated with epilepsy in children with CP. Our integrated metagenomic and metabolomic analyses have shed mechanistic insights on the potential roles of *B. fragilis* and *D. invisus* in neuroprotection. The integrated analyses have also provided evidence for the involvement of the GMBA in epilepsy-related dysbiosis of kynurenine, serotonin, and dopamine pathways and highlighted their complex interplay with neuroimmune and neuroendocrinological pathways. We have identified promising microbiome targets for future validation studies and potential microbiome-based interventions for pediatric epilepsy.

## Data availability statement

The datasets presented in this study can be found in online repositories. The names of the repository/repositories and accession number(s) can be found at: https://www.ebi.ac.uk/ena, PRJEB51713, https://figshare.com/, doi.org/10.6084/m9.figshare.19380527.v1.

## Ethics statement

The studies involving human participants were reviewed and approved by Hong Kong West Cluster Institutional Review Board (UW 21-029). Written informed consent to participate in this study was provided by the participants' legal guardian/next of kin.

## Author contributions

HT and SC conceived the study. AC and VL recruited the subjects, collected the samples, and phenotypic and clinical data. YP processed the samples and performed bioinformatic analyses. AC, YP, and VL wrote the manuscript. HT and SC critically revised the manuscript. All authors contributed to data interpretation and have read and approved the final article.
