# Supplementary material for: The role of the gut-microbiome-brain axis in metabolic remodeling amongst children with cerebral palsy and epilepsy
Source: Front Neurol. 2023 Feb 27;14:1109469. doi: 10.3389/fneur.2023.1109469 (PMC10009533; doi:10.3389/fneur.2023.1109469)
Supplement: Supplementary file 1 [file Table_1.pdf]

Supplementary Tables

Table S1. Associations between environmental factors and overall microbial compositions.

Factor	R^2	*P* value
Patient group		
CPE/NECP	0.05	0.31
NECP/DSE/DRE	0.11	0.233
Age	0.06	0.235
Sex	0.05	0.555
Type of cerebral palsy	0.15	0.515
GMFCS class	0.21	0.302
Number of AEDs used (at the time of enrollment)	0.04	0.664
Enzyme inhibitors use	0.07	0.117
Bowel habit	0.04	0.735
Bristol scale (7)	0.03	0.914
Probiotics use	0.07	0.157
7-day dietary intake (number of portions consumed per week) ¶		
Cereal	0.05	0.313
Vegetables	0.07	0.08
Fruit	0.04	0.842
Meat/poultry	0.04	0.763
Egg products	0.05	0.373
Fish	0.05	0.423
Other seafood	0.04	0.704
Bean/nut products	0.07	0.109
Dairy products	0.04	0.699
Sugary drinks	0.05	0.374

GMFCS: Gross Motor Function Classification System; AED: Antiepileptics; DRE: Drug resistant epilepsy; DSE: Drug sensitive epilepsy; GI: Gastrointestinal medications including laxatives, antacids, etc.

¶ Total size of food, calculated by frequency × size per meal.

P values were given by permutational multivariate analysis of variance (PERMANOVA).

Table S2. Microbial metabolic pathways potentially different between DRE and DSE in CP patients.

	Median (IQR) abundance			Prevalence			GLM modeling	
	DSE	DRE	*P* value	DRE	DSE	*P* value	Adjusted coefficient	*P* value
Histamine degradation	0e+00 (0e+00, 0e+00)	7.22e-07 (0e+00, 3.59e-06)	**0.089**	12.5%	60.0%	0.217	1.2 (1.05, 1.38)	**0.023**
Dopamine degradation	0e+00 (0e+00, 0e+00)	7.22e-07 (0e+00, 3.59e-06)	**0.089**	12.5%	60.0%	0.217	1.2 (1.05, 1.38)	**0.023**
DOPAC synthesis	0e+00 (0e+00, 0e+00)	1.92e-04 (0e+00, 2.35e-04)	**0.060**	12.5%	60.0%	0.217	1.07 (1.01, 1.13)	0.054
Alanine degradation II	1.45e-05 (7.55e-06, 2.06e-05)	4.06e-05 (2.41e-05, 9.56e-05)	**0.030**	87.5%	100.0%	1.000	1.1 (0.95, 1.28)	0.237
Lysine degradation II	2.21e-07 (0e+00, 5.52e-06)	7.81e-06 (3.84e-06, 1.58e-05)	**0.064**	50.0%	100.0%	0.105	1.14 (1.04, 1.25)	**0.020**

GLM: generalized linear model. Models were adjusted for sex.
