# Supplementary figures and images for: The role of the gut-microbiome-brain axis in metabolic remodeling amongst children with cerebral palsy and epilepsy
Source: Front Neurol. 2023 Feb 27;14:1109469. doi: 10.3389/fneur.2023.1109469 (PMC10009533; doi:10.3389/fneur.2023.1109469)

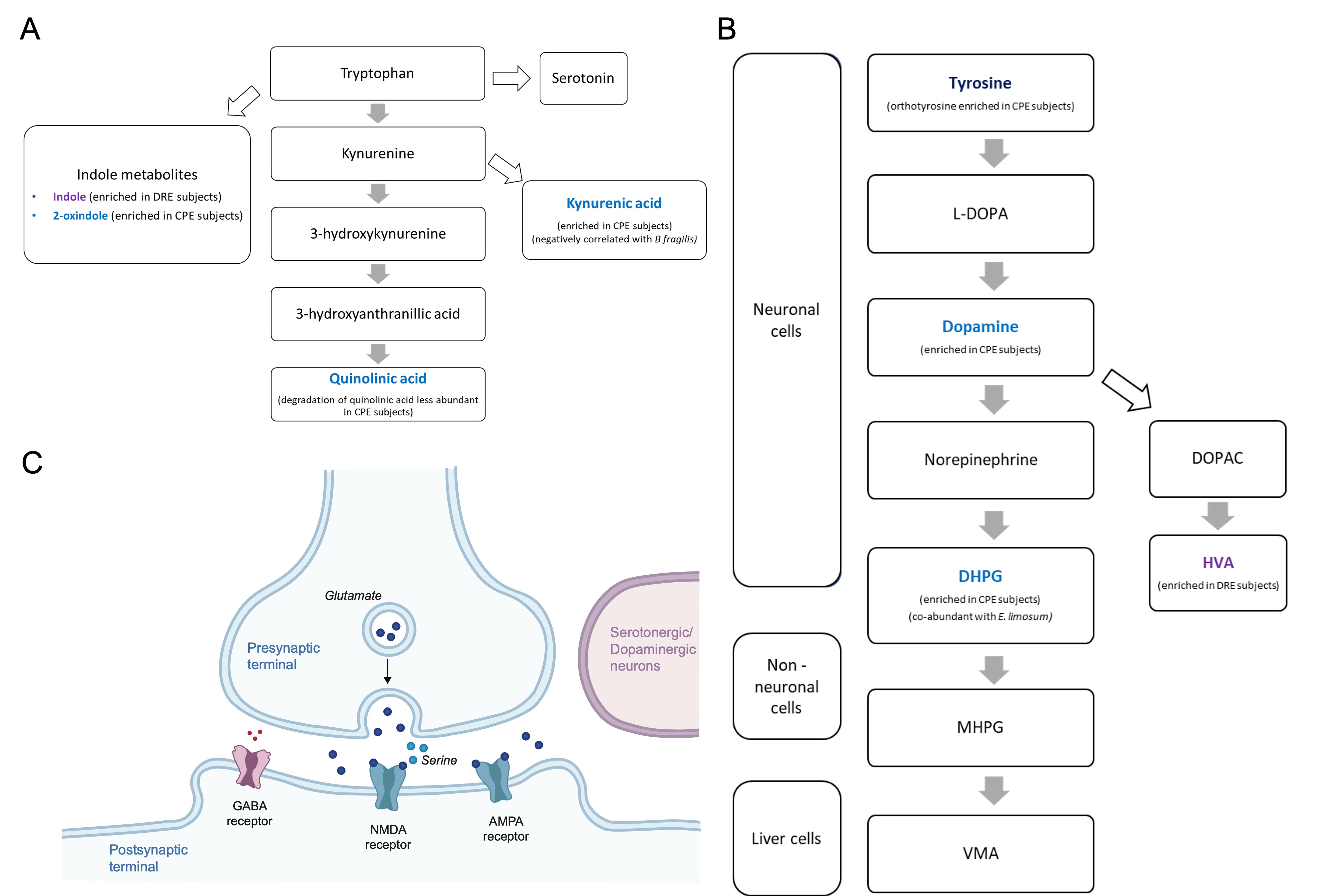

Supplement: Supplementary file 2 [file Image_1.jpg]
